# The Applications of Cone-Beam Computed Tomography in Endodontics: A Review of Literature

**Published:** 2014-12-24

**Authors:** Amir Hosein Kiarudi, Mohammad Jafar Eghbal, Yaser Safi, Mohammad Mehdi Aghdasi, Mahta Fazlyab

**Affiliations:** a * Oral and Maxillofacial Radiology Department, Dental School, Shahid Beheshti University of Medical Sciences, Tehran, Iran; *; b *Iranian Center for Endodontic Research, Research Institute of Dental sciences, Shahid Beheshti University of Medical Sciences, Tehran, Iran*

**Keywords:** Cone-Beam Computed Tomography, CT Scan, Endodontics, Three-dimensional Imaging

## Abstract

By producing undistorted three-dimensional images of the area under examination, cone-beam computed tomography (CBCT) systems have met many of the limitations of conventional radiography. These systems produce images with small field of view at low radiation doses with adequate spatial resolution that are suitable for many applications in endodontics from diagnosis to treatment and follow-up. This review article comprehensively assembles all the data from literature regarding the potential applications of CBCT in endodontics.

## Introduction

In 1899 Kells brought a new era to dentistry and more specifically endodontics, by stating the possibility of detecting a lead wire placed in the root canal on a “radiogram” that would enable establishing the length of a root canal [1, 2]. Since then conventional radiography has been a fundamental tool in endodontic practice [3].

Successful management of endodontic problems depends on diagnostic imaging techniques to provide the critical information about the teeth under examination, and their surrounding anatomy [4]. Therefore, radiographic examination is a crucial component in management of endodontic problems. It comprises a basis for all aspects of endodontic treatment from diagnosis and treatment planning to outcome assessment [5]. Conventional radiography has remained the foundation of imaging in endodontics. However, in recent decades, modern techniques of medical imaging have also been successfully utilized in the various fields of dentistry [4].

Since the first efforts by the pioneers trying to apply the conventional computed tomography (CT) and micro-CT in endodontics, the introduction of maxillofacial cone-beam computed tomography (CBCT) in 1996 provided the first clinically and practically applicable technology demonstrating the application of three-dimensional (3D) imaging for endodontic considerations [3, 6]. After approval of Food and Drug Administration (FDA), dentists have welcomed the advantages of CBCT 3D imaging technology over conventional radiography. By providing true 3D images at a lower cost than conventional CT, CBCT may indeed be the next major advancement in dentoalveolar imaging, with radiation risks similar to current methods of intraoral imaging, including full-mouth and panoramic radiographic examination [7, 8].

This article tends to review the fundamentals of CBCT and also its applications in contemporary endodontic practice.

**Role of imaging in endodontics**

X-ray imaging serves at all stages of endodontics from diagnosis of odontogenic and nonodontogenic pathoses to treatment of the root canal system in a compromised tooth, biomechanical instrumentation, obturation, and healing assessment [3]. Intraoral periapical radiographs during endodontic procedures are still the most commonly used treatment adjuncts. They provide useful information for the presence and location of periradicular lesions, root canal anatomy and the proximity of adjacent anatomical structures [9]. They are used for preoperative, intraoperative and postoperative assessment and follow-up. Despite many applications in endodontics, there are still many shortcomings that can be named for periapical X-ray imaging. As a result of superimposition, periapical radiographs reveal limited aspects of the 3D anatomy thus the amount of information gained from conventional film and digitally captured periapical radiographs is limited [5]. Several factors can result in the reduced diagnostic ability of conventional radiography [9] which are discussed below.

**Figure 1:**
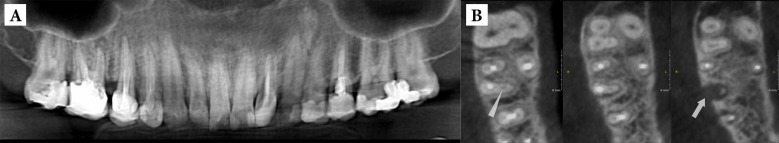
*A)* A panoramic image of a patient complaining of dull pain two years after root canal therapy (RCT) of the right maxillary first molar. Note the apical periodontitis around the apex of the MB root; *B)* Axial CBCT scanning of the maxillary right quadrant showing the undetected and untreated second mesiobuccal canal (MB2) (arrow head)

***1. Compression of 3D structures:*** An accurate assessment of the spatial relationship of root(s) to the surrounding anatomy and any associated periapical lucency is often precluded by the compression of 3D anatomy associated with conventional radiography [10]. In addition, locating the lesions within the target root (*e.g.* root resorption) may be difficult [11, 12]. Moreover, if more accurate imaging is not used, anatomical complexities and diseases affecting the dental hard tissues, such as resorption [13] and operative procedural errors [14], may remain undetected. As a result, the accuracy of diagnosis is subsequently reduced [15, 16]. In parallax radiographic images, altering the horizontal angulation of the X-ray beam, have been shown to improve the depth of perception and determination of the spatial relationship between tooth and alveolar structures [17]. Several intraoral views taken at different angles may be essential for diagnosing traumatic dental injuries (*e.g.* root fractures, luxation and avulsion injuries) [18, 19]. It should be noted that the identification of all relevant anatomic varieties or diseases is not guaranteed by multiple intra-oral radiographs [20, 21].

***2. Geometric distortion: ***Radiographic images do not always accurately replicate the area of interest, because of the structural complexity of the maxillofacial area [22]. Intraoral parallel periapical radiographs provide a more accurate geometric representation of the object of interest than images taken by “bisecting angle” technique [23-25]. To achieve paralleled images, the image receptor should be positioned parallel to the tooth under examination, and the X-ray beam should be perpendicular to both [26]. Despite the availability of paralleling devices, the anatomical confine of the oral cavity makes their use challenging. Even when the paralleling technique is perfectly fulfilled, a minimum magnification of 5% can be expected in the final image [27].

Positioning the image receptor parallel to the long axis of the tooth may be achievable with teeth that have relatively straight roots (*e.g.* incisors and premolars). However, root curvature is not uncommon in multi-rooted teeth. In these situations, it is impossible to completely eliminate the geometric distortion and magnification. The net result is that diverging roots (particularly relevant in the posterior maxilla) will not be shown accurately in a single exposure because of varying degrees of distortion [28].

***3. Anatomical noise:*** The problem of anatomical noise in endodontics was first detected by Brynolf who stated that the projection of the incisive canal over the apices of maxillary incisors may complicate the radiographic interpretation [17, 29]. Anatomical features such as overlying anatomy, the thickness of the cancellous bone and cortical plate and finally the relationship of the root apices to the cortical plate [9], can make interpreting images difficult, because they may obscure the area of interest [30, 31]. These may include radiolucent (*e.g.* incisive foramen, maxillary sinus) or radiopaque (*e.g.* zygomatic prominence) structures [9]. Anatomical noises are described as complicating factors in the accurate detection of periapical lesions [32-34] and external root resorptions (ERR) [35, 36]. In their presence, the reduction in contrast will be greater within the area of interest [30, 31, 37].

**Cone-beam computed tomography (CBCT)**

Where 3D imaging is necessary, CBCT is considered the standard of care by some authors [38-42]. Although it is originated from conventional medical CT, CBCT differs from the CT in a number of fundamental ways which improve its suitability for dental imaging [4]. In the late 1990s, two independent Italian and Japanese groups developed a new tomographic scanner known as “CBCT” or “digital volume tomography (DVT)” specifically for maxillofacial and dental uses [5, 43, 44]. Offering the advantage of lower radiation dose [45, 46], CBCT has been applied for oral and maxillofacial surgery, implantology, endodontics, orthodontics, periodontics and temporomandibular disorders (TMD) [5, 26, 47].

**Figure 2:**
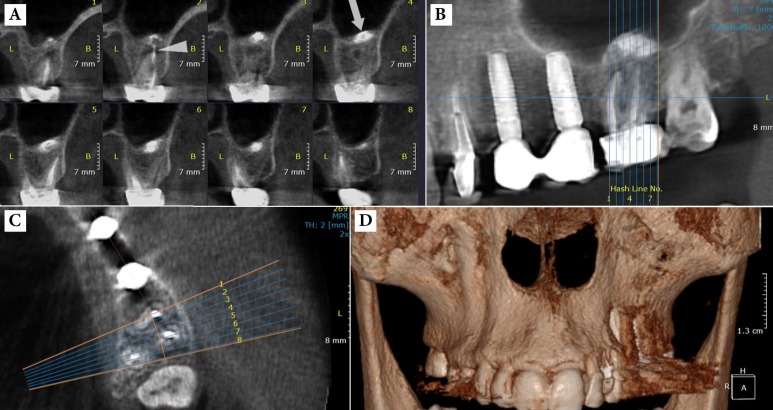
*A*) Cross-sectional CBCT view showing the extrusion of sealer after root canal therapy (RCT) of the left maxillary first molar. This image also represents the anatomical relation of roots and maxillary sinus; *B*) Note the extrusion of the sealer through the periapical lesion into the maxillary sinus. *C*) Anatomical relation of roots and buccal/palatal cortical plates *D*) Three-dimensional reconstruction

As the name shows, CBCT has cone-shaped X-ray beam that captures a cylindrical or spherical volume of data, described as the field of view [5]. A 3D volume of data is acquired with a single sweep of the scanner, using a simple and direct relationship between beam source and sensor; the latter rotating 180-360^°^ around the patient’s head [5]. During the exposure sequence, hundreds of planar projection images are obtained from the field of view (FOV), in an arc of at least 180^°^. In this way CBCT presents precise, essentially accurate and immediate 3D radiographic images. Only one rotational sequence of the gantry is necessary to acquire enough data for image reconstruction, as CBCT exposure incorporates the entire FOV [38, 39]. Each projection image is consisted of up to of 512^2^ pixels. In addition, the reconstructed 3D data set will comprise of 512^3^ 3D pixels, or voxels [4]. It is possible to increase number of pixels per matrix (projection image) from 512^2^ to 1024^2^ which also increases the resolution. However, this improved resolution is gained at the cost of increasing radiation exposure by 2 to 3 folds [48]. Also, the scan time typically has a range from 10 to 40 seconds, depending on the equipment and exposure parameters employed. However, many CBCT systems utilize a pulsatile X-ray beam. Consequently, with these systems, the actual patient exposure time can be as low as 2 to 5 seconds [45].

CBCT scanners use simpler, less complicated and less expensive hardware than CT scanners [49, 50], which means that the cost of a CBCT scanner is significantly less than a CT scanner. This has resulted in an increase in its application in dental practices [40, 51]. CBCT causes fundamental changes in diagnosis and management of endodontic problems. The clinician can easily apply a simple software to evaluate the areas of interest in any plane [45].

***Classification of CBCT***

CBCT systems are most commonly classified in accordance with the scan volume or dimensions of their FOV, which are primarily reliant on the detector size and shape, beam projection geometry and the ability to collimate the beam. As mentioned earlier, the shape of the FOV can be either cylindrical or spherical. Collimation of the primary X-ray beam limits the radiation exposure to the region of interest. Therefore, the limitation of field size ensures that an optimal FOV can be selected based on disease presentation and the region of interest to be imaged for each patient. Based on available or selected scan volume height, the use of units can be classified as follows: **Small volume or localized region;** also called as focused, small field, limited field or limited volume, **Single arch;** CBCT scans have a FOV height ranging from 5-7 cm within one arch, **Inter arch;** CBCT scans have a FOV height ranging from 7-10 cm, **Maxillofacial;** CBCT scans have a FOV height ranging from of 10-15 cm and **Craniofacial;** CBCTs have a FOV height greater than 15 cm [3].

**Figure 3:**
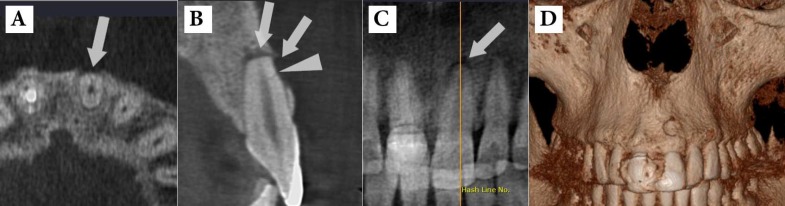
*A)* CBCT view shows a tiny horizontal root fracture on the buccal surface of the maxillary left central incisor caused by impact trauma; *B and C) *Note the two separate periradicular lesions in the apical area (arrow) and adjacent to the fracture line (arrow head) due to tooth necrosis; *D*) Three-dimensional reconstruction of the lesion in the periradicular buccal area

In general, the smaller scan volume causes the higher spatial resolution of the image. It is favorable that the optimal resolution of any CBCT imaging system used in endodontics does not exceed the average width of the periodontal ligament space (200 μm), considering the the earliest sign of periapical pathology being the discontinuity in the lamina dura and widening of the periodontal ligament space [7].

In addition to reducing capital costs, CBCT units with small FOV offer many advantages in endodontics. First, a small FOV means that high resolution images with a spatial resolution as low as 0.076 mm isotropic voxel size can be acquired at very low exposure dose. Also the image is taken without extensive reconstruction times that would be required with larger FOV systems due to the greater file sizes to be processed. Second, a limited FOV reduces the volume examined that the practitioner is responsible to interpret. CBCT systems are also classified by less popular methods based on the patient position [4].

***Effective dose of CBCT***

Comparing the radiation dose of different CBCT scanners with medical CT scanners may be confusing due to different units of radiation dose that can be used [5]. Therefore, radiation exposures are converted to effective dose which is measured in Sieverts (Sv), for a meaningful comparison of radiation risk. The Sv is a large unit, so in maxillofacial imaging milliSieverts (mSv) [10^-3^] or microSieverts (μSv) [10^-6^] are presented. The radiation dose to specific tissues is measured and adjusted for the amount of that tissue in the FOV, or weighted in accordance with radiation sensitivity of the tissue. Then the weighted tissue/organ doses are summed to compute effective dose. Comparisons can be performed according to natural background radiation [3].

There are a number of factors affecting the radiation dose produced by a given CBCT system. The nature of the X-ray beam (whether it is continuous or pulsatile), the degree of rotation of the X-ray source and detector and the size of the FOV all depend on the radiation dose. The amount and type of beam filtration and the exposure parameters naming kilovoltage (kVp), milliamper (mA) and voxel size should be also added to the list. Some exposure parameters such as beam filtration, the nature of the X-ray beam and to some extent, the FOV, are specific to a particular system, while other factors such as the degree of X-ray source rotation, kVp and mA are changeable on most systems [4].

***Advantages of CBCT***

As mentioned earlier, CBCT overcomes the limitations of conventional radiography by producing 3D images that allow a comprehensive appreciation of the anatomy, and the spatial relationship of the pathosis and anatomical structures [52].

The clinician can choose and view slices of the volumetric data in all the orthogonal planes and in non-orthogonal planes. Therefore, anatomical noise can be easily eliminated [48]. CBCT voxels are isotropic, so they ensure that the produced images are geometrically accurate and image measurements, in any plane, are free from distortion [40]. The 3D geometric accuracy of CBCT has been shown repeatedly [53, 54]. In contrast to CBCT, CT images are comprised of anisotropic voxels, which limit the geometric accuracy of this form of imaging [45].

However, the main advantages of CBCT over CT are the reduced patient exposure to ionizing radiation [43, 44] and a superior image quality with regard to dental hard tissues [55-57] and bone assessment [58]. As the CBCT X-ray beam is pulsatile, the patient is often exposed to radiation for only a small portion of the overall scan time. In addition, the X-ray source can be collimated so that the radiation is limited to the area of interest. This produces a specific volume of data (FOV) appropriate and relevant to the patient’s needs. The smaller the FOV, the less the radiation exposure to the patient [59]. As mentioned, the degree of rotation of the X-ray source around the patients head can also be altered. The higher degrees of rotation produce higher number of images [3]. However, this may be associated with an increased diagnostic yield, but at the expense of greater radiation exposure to the patient. The scan times attainable with CBCT are short and comparable with panoramic radiography. This is helpful in that the likelihood of patient movement during the scan is less. Furthermore, as previously stated, the CBCT hardware is much smaller and less expensive than CT machines. So, CBCT is well suited for application in dental practice [45].

**Figure 4:**
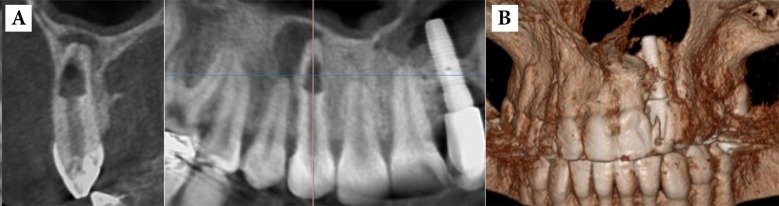
*A)* Internal root resorption in the maxillary right canine: note the extensive bone resorption adjacent to the perforated root site and apical periodontitis around the apical foramen; *B*) Three-dimensional reconstruction of the region

Perhaps the most important advantage of CBCT in endodontics is three dimensional demonstrations of the anatomic features. CBCT units reconstruct the projection data to produce images in three orthogonal planes (axial, sagittal, and coronal) [3].

***Limitations of CBCT***

At present, the spatial resolution and the contrast resolution of CBCT is lower than that of conventional film-based or digital intraoral radiography [60]. Radiographic artifacts are another problem in CBCT imaging. When the CBCT X-ray beam encounters a very high density object, such as enamel or metallic restorations, lower energy photons in the beam are absorbed by the structure. As a result, the mean energy of the X-ray beam increases [48], which is referred to “beam hardening”. This produces two types of artifact that can reduce the diagnostic yield of the images: distortion of metallic structures, called “cupping artifact” and the appearance of streaks and dark bands between two dense structures [48], so that these artifacts may reduce the diagnostic yield of images [28, 61]. In addition, the patient have to stay absolutely still [45] as his/her movement can adversely affect the sharpness of the final image during the scan [48].

**Applications of CBCT in endodontics**

The potential benefits of CBCT in endodontics are vast especially where the anatomy being assessed is complex [40, 45]. However, the higher effective dose of ionizing radiation in comparison with conventional two-dimensional radiographs is not justifiable in every case. Generally, the application of CBCT in endodontics should be limited to the evaluation and management of complex endodontic conditions which will be named here.

***A. Detection of apical periodontitis***

The most common pathologic conditions affecting the teeth are the inflammatory lesions of the pulp and periapical areas [3]. In this regard CBCT is significantly more accurate and sensitive than conventional radiography in the identification of apical periodontitis in humans [62]; periapical bone destruction associated with endodontic infection can be identified using CBCT before the evidence of their existence becomes identifiable on conventional radiographs [63, 64]. Although there were considerable disagreements between CBCT and periapical radiographs for assessing the periapical status of molar teeth, especially for the maxillary arch [65], CBCT detected the periapical lesions 62% more than conventional radiographs, and even the assessment of the subject teeth was increased by parallax views in the latter technique [28, 66]. In addition, CBCT can demonstrate bone defects of the cancellous bone and cortical bone separately. As a result, the identification of apical periodontitis was substantially higher with CBCT than with periapical radiography [61]. Also CBCT presented significantly more findings, such as expansion of lesions into the maxillary sinus, sinus membrane thickening and missed canals. Patel *et al.* [67] used an *in vitro *model consisting of 2 mm diameter defects placed in the cancellous bone at the apices of 10 first molar teeth on six partially dentate intact human dry mandibles. They reported a detection rate of 24.8% and 100% for intraoral radiography and CBCT imaging, respectively. Thus, CBCT is found to be a more sensitive diagnostic method for detecting apical periodontitis (Figure 1).

***B. Assessment of Potential surgical sites***

CBCT is an extremely useful tool in the planning of surgical endodontic treatment [68, 69]. The spatial relationship of the specific tooth root(s) undergoing the surgical procedure (and the associated bony destruction) to adjacent anatomical structures such as the maxillary sinuses, the inferior dental nerve canal and the mental foramen can be precisely assessed [28]. Rigolone *et al.* [68] concluded that CBCT may play an important role in planning for periapical microsurgery on the palatal roots of maxillary first molars. The presence or absence of the maxillary sinus between the roots could be presented, and the distance between the cortical plate and the palatal root apex could be measured (Figure 2).

**Figure 5:**
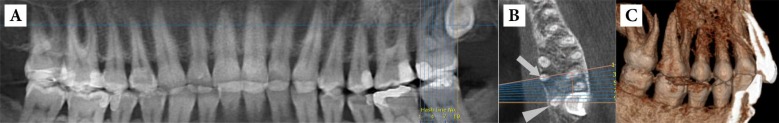
*A*) Panoramic view of a patient complaining of pain in the upper left quadrant: the second molar has a normal appearance. *B*) The axial view showing the abnormal anatomy of the second molar with four roots. *C*) Three dimensional reconstruction of the alveoli showing the two separate palatal roots of maxillary left second molar

***C. Assessment of traumatic dental injuries***

CBCT provides valuable information regarding the detection of type and severity of traumatic dental injuries [11]. In the literature, the advantages of CBCT have been emphasized in the assessment and management of dentoalveolar trauma [11, 49]. In addition, CBCT has been shown to be much more sensitive in detection of horizontal root fractures than multiple periapical radiographs [70, 71]. By eliminating anatomical noise and image compression, the extent of the injuries to the teeth and the alveolar bone can be assessed accurately which allows appropriate treatment to be assuredly considered. For instance, the degree and direction of displacement related to luxation injuries can be assessed easily applying CBCT [72].

Small volume CBCT scanners capture all teeth and surrounding anatomy in a 4×4 cm FOV. Therefore, in a single scan, multiple teeth can be assessed without geometric distortion. Furthermore, when CBCT is indicated as an extraoral imaging modality, patient comfort is increased during the imaging process. Where patient has difficulty in accommodating bulky film holders and image receptors or conventional imaging is intensified by potentially mobile teeth and painful oral and dental tissues, CBCT is particularly important in the appraisal of dental injuries [4] (Figure 3).

***D. Diagnosis of different types of root resorption***

After dental luxation and avulsion injuries, external root resorption (ERR) is a common complication [73, 74]. The sensitivity of conventional radiography is considerably poorer than CBCT in the detection of ERR in its early stages, and before the resorption becomes evident on conventional radiographs, significant hard tissue damage may have potentially occurred to the affected tooth. Furthermore, it must be noted that when a diagnosis of root resorption is made based on conventional radiographic findings, ERR superimposed on the root canal may mimic internal resorption [75] and differentiation between external cervical resorption (ECR) and internal resorption can be particularly difficult [75, 76]. There are several cases illustrating the application of CBCT in detection of small lesions, localizing and differentiation of the ERR from other conditions, classification of the lesion, and determining the prognosis and treatment [11, 49, 77-79]. The resorptive lesion is unnoticed, until it becomes evident on conventional images, and consequently significant damage may already have occurred to the tooth. At present CBCT is often applied to assess the extent of certain types of ERR and the prognosis of the affected tooth [80] (Figure 4).

***E. Assessment of Root canal anatomy and morphology***

The success of endodontic treatment depends on identification, cleaning, shaping and obturation of all accessible areas of root canal system [81-83]. As a result, failure to distinguish and treat all canals can negatively affect treatment outcome [84]. For example, the prevalence of a second mesiobuccal canal (MB2) in maxillary first molars has been reported to be 69% to 93% depending on the study method. This variability occurs in the buccolingual plane because of superimposition of anatomic structures [16, 85]. Conventional radiographs, at their best, can only reveal up to 55% of these configurations [86]. In contrast, with increasing resolution of CBCT, the detection rate enhanced from 60% to 93.3% [45] (Figure 1).

To accurately assess the degree of curvatures associated with the roots of teeth, CBCT is a reliable tool [87], and the preoperative availability of this information reduces the chances of occurring the aberrations outlined above. Furthermore, when endodontic treatment for teeth with anatomical and morphological anomalies such as dens invaginatus and tooth fusion, is required, CBCT has been shown to be a useful assessment and treatment planning tool [88, 89] (Figure 5).

***F. Diagnosis of vertical root fractures***

Root fractures are difficult to diagnose accurately using conventional radiography while they are less common than fractures of the crown and account for only 7% of dental injuries [3, 90]. Detecting the presence of vertical root fractures (VRF) is an often dilemma in endodontics [91]. Clinical and radiographic sign of the presence of root fracture does not always present itself until the fracture has been occurred for some time. While a deep, isolated, thin periodontal pocket is suggestive of VRF, however, even clinical signs of longstanding VRF maybe little more than a draining buccal sinus, which is definitely not pathognomonic of the problem. It should be noted that radiographic appearances suggestive of VRF such as J-shaped and halo-shaped radiolucencies do not appear until considerable bone destruction has occurred [92] and similar shaped radiolucencies may occur in cases of apical periodontitis not associated with VRF [4]. Studies have shown that CBCT is more sensitive than conventional radiography in identification of VRF [93-95]. Small-FOV CBCTs should be used for representing VRFs of endodontically treated teeth [96]. However, because scatter produced by the root filling or other high-density intraradicular materials may incorrectly suggest the presence of a fracture, it should be taken into consideration when assessing root filled teeth for VRF using CBCT [94].

## Conclusion

Studies demonstrate the advantages of CBCT over conventional imaging for almost all endodontic applications, with the exception of assessing the quality of root canal fillings. It is clear that the usefulness of the CBCT cannot be disputed. Of course, availability, dose and costs must be considered when prescribing CBCT imaging for the patient.
